# Variation in diagnostic performance of fecal immunochemical test (FIT) by cutoffs used in screening programs globally

**DOI:** 10.1016/j.eclinm.2026.103835

**Published:** 2026-03-16

**Authors:** Idris Ola, Teresa Seum, Sigrid V. Carlsson, Michael Hoffmeister, Hermann Brenner

**Affiliations:** a Division of Clinical Epidemiology of Early Cancer Detection, German Cancer Research Center (DKFZ), Heidelberg, Germany; b Heidelberg Medical Faculty, Heidelberg University, Heidelberg, Germany; c Department of Translational Medicine, Division of Urological Cancers, Medical Faculty, Lund University, Lund, Sweden; d Cancer Prevention Graduate School, German Cancer Research Center (DKFZ), Heidelberg, Germany

**Keywords:** Colorectal cancer screening, FIT thresholds, Colonoscopy, Stool-based test, Sensitivity, Specificity

## Abstract

**Background:**

Quantitative fecal immunochemical test (FIT) is widely used for colorectal cancer (CRC) screening, with substantial variation in positivity thresholds across countries. This study examines how these thresholds affect FIT's diagnostic performance for detecting CRC and advanced precancerous lesions (APCL).

**Methods:**

We analyzed data from a single round screening of 7398 men and women aged 50–79 years, enrolled between 2008 and 2020 in the German BLITZ study, who provided a stool sample for FIT prior to screening colonoscopy. Using FIT positivity thresholds from 30 countries across five continents, we estimated positivity rates, sensitivities for CRC, APCL, and any advanced neoplasia (AN), as well as specificity for no AN, with colonoscopy as the reference standard. Subgroup analyses were conducted by age and sex.

**Findings:**

FIT positivity thresholds across the included countries ranged from 8.5 (Belgium, Flanders) to 120 μg Hb/g feces (England, Wales and Northern Ireland). This wide range went along with very wide ranges of positivity rates and diagnostic performance parameters: positivity rate, 17.6% (95% CI: 16.7–18.5) to 2.6% (95% CI: 2.3–3.0); sensitivity for CRC, 98.1% (95% CI: 90.1–100.0) to 55.6% (41.4–69.1); sensitivity for APCL, 45.5% (95% CI: 41.9–49.0) to 12.2% (95% CI: 10.0–14.7); sensitivity for any AN, 48.9% (95% CI: 45.4–52.4) to 15.0% (95% CI: 12.7–17.7); specificity for no AN, 86.3% (95% CI: 85.5–87.2) to 98.9% (95% CI: 98.7–99.2).

**Interpretation:**

The broad range of FIT positivity thresholds across countries goes along with very different diagnostic performance parameters. While this limits comparability of results between countries, our results underscore the potential of FIT as a flexible screening tool that can be adapted to the evolving healthcare system capacities in different countries.

**Funding:**

German Research Council, the 10.13039/501100002347Federal Ministry of Education and Research, and the 10.13039/501100005972German Cancer Aid.

**Research in context**

**Evidence before this study**

We searched PubMed for articles published up to September 30, 2025, reporting on the diagnostic performance of fecal immunochemical tests (FIT) for colorectal cancer and advanced colorectal neoplasia based on various positivity cut-offs used in different countries. We used the following search terms in PubMed: (“fecal immunochemical test” OR “faecal immunochemical test” OR “FIT” OR “fecal occult blood test” OR “FOBT” OR “immunochemical fecal occult blood test”) AND (“sensitivity and specificity” OR “predictive value of tests” OR “diagnostic performance” OR “sensitivity” OR “specificity” OR “positive predictive value”) AND (“cutoff” OR “cut-off” OR “threshold” OR “positivity threshold” OR “positivity rate” OR “quantitative cutoff” OR “variation” OR “international comparison” OR “screening program” OR “global”) AND (“colorectal cancer” OR “colorectal neoplasms”). Evidence shows that fecal immunochemical tests are widely used in colorectal cancer screening, but screening programs in different countries set very different thresholds for a positive result. In addition, existing studies mostly relied on relative estimates of FIT diagnostic performance across varying thresholds, given that colonoscopy results were mostly available for FIT positive participants only.

**Added value of this study**

Drawing on FIT thresholds from 30 countries in five continents, this study, which is based on a large screening colonoscopy cohort with colonoscopy results available for all participants regardless of FIT results, provides absolute estimates of FIT diagnostic metrics. It also demonstrates how variation in FIT thresholds translates into substantial variability in positivity rates and diagnostic performance for detecting colorectal cancer and advanced precancerous lesions. Our clinical impact modeling further demonstrates the real-world implications of these findings by showing the trade-off between early cancer detection and the availability of colonoscopy resources in various countries.

**Implications of all the available evidence**

The large variation in FIT performance across different thresholds demonstrates its adaptability as a screening tool. This variability provides a foundation for further research on optimizing FIT cutoffs to meet differing public health needs and capacities. Overall, our findings show how strongly healthcare system decisions about FIT thresholds affect detection rates of colorectal cancer and its precursors. Our results may help policymakers and practitioners to select thresholds that align with available resources and screening goals in FIT-based programs.

## Introduction

Fecal immunochemical test (FIT) is the most widely used screening test for colorectal cancer (CRC) worldwide, and quantitative FIT has formed the cornerstone of many population-based organized CRC screening programs, especially in Europe and Asia.^1^^,^^2^

Being a non-invasive test that quantifies occult hemoglobin in stool, its diagnostic performance is heavily influenced by the hemoglobin concentration cut-off level used to define a positive test result. This threshold determines the balance between sensitivity (the ability to detect true positive cases) and specificity (the ability to exclude false positive cases). Therefore, a lower cut-off level increases sensitivity, improving the likelihood of detecting CRC and advanced precancerous lesions (APCL), but often at the expense of reduced specificity, potentially leading to higher false-positive rates and unnecessary follow-up colonoscopies.^3^ Conversely, a higher cut-off level improves specificity but risks missing cases that could benefit from early intervention.

A review of national and regional CRC screening programs globally shows a wide variation in adopted FIT cut-off levels, with existing cut-offs in use ranging from as low as 8.5 μg hemoglobin per gram feces (μg Hb/g) in Belgium to as high as 120 μg Hb/g in the United Kingdom.^3^ Since a positive FIT result requires follow-on colonoscopy for definitive diagnosis, these differences essentially reflect variations in capacity for colonoscopy service, specifically the availability of equipment and specialist personnel, as well as differences in screening objectives, resource availability, and the overall healthcare system capacity to manage prevalent cases.^4^^,^^5^ However, the large variation in FIT thresholds in real-world screening contexts highlights the need to understand the implications of these variations on FIT performance. While the relationship between FIT thresholds and diagnostic performance is well-established, most previous studies have been conducted in settings in which colonoscopy results were available for FIT positive participants only, which prohibited calculation of absolute sensitivities and specificities and evaluation of test performance below the specific FIT cutoff used in the respective program.^6^^,^^7^ A comprehensive, standardized comparison of FIT performance across cut-offs currently used in organized screening programs worldwide is still lacking.

This study, therefore, systematically evaluates the diagnostic performance of FIT across the range of thresholds used in different countries, providing evidence of the trade-offs between sensitivity and specificity inherent to each cut-off and highlighting the implications for overall detection rates of CRC and APCL.

## Methods

### Study design and data collection

We conducted a diagnostic performance study using data from a single screening round in the German “Begleitende Evaluierung Innovativer Testverfahren zur Darmkrebsfrüherkennung” (BLITZ) study. Details of the design of the BLITZ study have been provided in previous reports.^8^, ^9^, ^10^ Briefly, BLITZ is an ongoing study among participants of screening colonoscopy that started in 2005, involving 20 gastroenterology practices in the south of Germany. After obtaining written informed consent, the study participants provide a stool sample for quantitative FIT prior to screening colonoscopy.

**Fig. 1:**
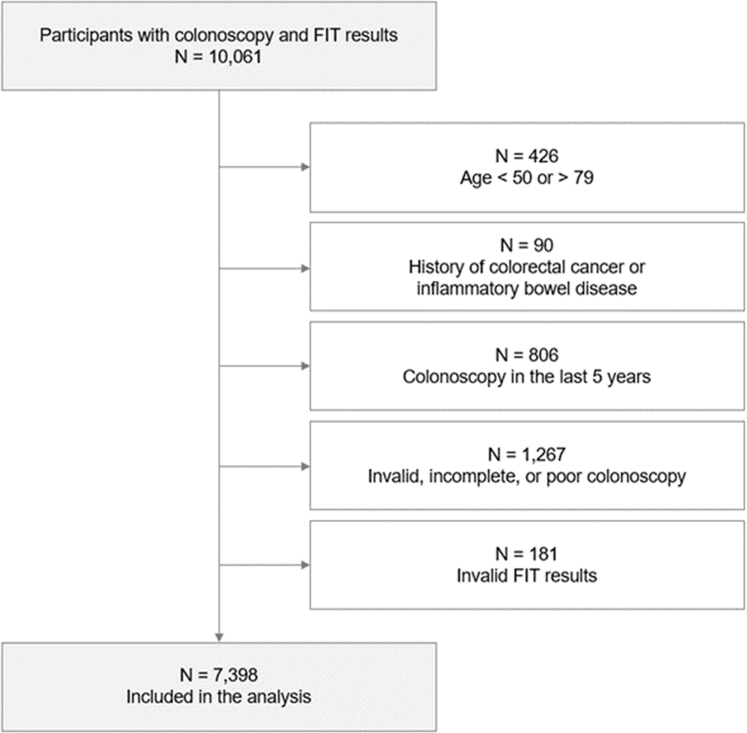
BLITZ flow diagram, inclusion and exclusion criteria. Abbreviation: FIT, fecal immunochemical test.

For this analysis, we included participants recruited from 2008 to 2020, when the same quantitative FIT (FOB Gold; Sentinel Diagnostics, Milano, Italy) was applied. Participants were excluded if they were younger than 50 years or older than 79 years, had a history of CRC or inflammatory bowel disease, underwent a colonoscopy within the past five years, had an invalid, incomplete, or poor colonoscopy (e.g., had inadequate bowel preparation or had polyps with unspecific features based on colonoscopy findings), or had invalid FIT results (e.g., providing stool samples after undergoing a colonoscopy). Fig. 1 provides the flowchart of patient selection for this study.

Between 2008 and 2012, stool samples were collected in small plastic containers, frozen at home and brought to the gastroenterology clinic on the day of colonoscopy, then stored at −20 °C until cold-chain transport to a central laboratory for analysis. From February 2012 onward, stool was collected using tubes with a hemoglobin-stabilizing buffer (10 mg stool in 1.7 mL buffer) and mailed directly to the German Cancer Research Center in Heidelberg, where they were refrigerated at 2–8 °C before centralized analysis. The FIT was processed on the Abbott Architect c8000 system. Both stool collection methods have been shown to yield comparable test results.^9^

Colonoscopy and histology reports were retrieved for all individuals undergoing screening colonoscopy at the collaborating practices (irrespective of FIT result), and relevant clinical and follow-up information was independently extracted by two trained research assistants, both blinded to the test outcomes.^8^, ^9^, ^10^

### Statistical analysis

The characteristics of the study population including findings at screening colonoscopy were described.

For evaluation of FIT diagnostic performance, we employed the cut-offs currently in use in various countries and regions that were identified in a recent global survey by Young et al.^3^ Based on these thresholds, we calculated the FIT sensitivity for CRC, APCL and any advanced neoplasia (AN, i.e., CRC or APCL) and specificity for no AN, using colonoscopy results as the gold standard. Advanced precancerous lesions (APCL) were defined as advanced adenomas and sessile serrated lesions 1 cm or larger in size, contained (tubulo-)villous features, or exhibited high-grade dysplasia.^11^

Subgroup analyses were conducted by age and sex to determine if and to what extent cut-off specific diagnostic performance parameters vary across demographics. Given the well-known variation of prevalence of advanced neoplasia by sex and age, we additionally calculated sex- and age-specific positive predictive values (PPVs) for the various cutoffs.

To illustrate the expected clinical implications of different thresholds at the population level, we further derived detection outcomes per 100,000 screened individuals, assuming the CRC and APCL prevalences observed in the BLITZ study. For each cut-off, the number of colonoscopies required, CRC, APCL, and AN detected or missed, as well as negative colonoscopies, were derived from the observed test performance parameters. Specifically, we calculated these quantities by applying the estimated sensitivity, specificity, and positivity rates at each FIT threshold to the observed prevalence of CRC, APCL, and AN in the BLITZ cohort. Missed cases were calculated as the difference between the total number of colonoscopy-confirmed cases and those detected by FIT. The number of positive and negative FIT results was determined using the estimated positivity rate, and the number of negative colonoscopies was calculated as the number of FIT-positive individuals without advanced neoplasia on colonoscopy.

All statistical analyses were conducted in R, using RStudio software, version 4.4.0 (RStudio, Inc., Boston, MA, USA).

### Ethics statement

The BLITZ study protocol was approved by the Institutional Review Boards of the Medical Faculty Heidelberg, University of Heidelberg (178/2005), and the medical chambers of the states of Baden-Württemberg (M118-05-f), Hesse (MC 254/2007), Rhineland-Palatinate (837.047.06 (5145), and Saarland (217/13).

### Role of the funding source

The funder of the study had no role in study design, data collection, data analysis, data interpretation, or writing of the report.

## Results

Main characteristics of the study population are shown in Table 1. A total of 7398 participants were included in the study with a slight female preponderance (51.6%, n = 3815). The mean age of participants was 61.5 (SD 6.6) years, with nearly half of the participants in the 50–59-year age group in both male and female participants. There were 824 (11.1%) participants with advanced neoplasia, comprising 54 (0.7%) with CRC and 770 (10.4%) with APCL. Prevalences of CRC and APCL were approximately 50% higher among male than among female participants.

**Table 1:** Characteristics of the included BLITZ study participants.

Characteristics	Total (n = 7398)	Women (n = 3815)	Men (n = 3583)
Age, y			
Mean (SD) [range]	61.5 (6.6) [50–79]	61.5 (6.5) [50–79]	61.6 (6.7) [50–79]
50–59, No. (%)	3605 (48.7)	1900 (49.8)	1705 (47.6)
60–79, No. (%)	3793 (51.3)	1915 (50.2)	1878 (52.4)
Most advanced finding at colonoscopy			
CRC, No. (%)	54 (0.7)	21 (0.6)	33 (0.9)
Advanced precancerous lesions, No. (%)^a^	770 (10.4)	314 (8.2)	456 (12.7)
Any advanced neoplasia, No. (%)^b^	824 (11.1)	335 (8.8)	489 (13.6)
No advanced neoplasia, No. (%)	6574 (88.9)	3480 (91.2)	3094 (86.4)

Abbreviations: CRC, colorectal cancer; SD, standard deviation; y, year; n, number.

a Defined as adenomas or sessile serrated polyps ≥1 cm, lesions with villous histologic features, or high-grade dysplasia.

b Includes CRC or APCL.

### Variation of diagnostic performance across FIT cutoffs used in different countries

Table 2 presents a summary of the FIT positivity thresholds in use at national, regional, or provincial levels in 30 countries spanning five continents globally. The thresholds ranged from 8.5 μg Hb/g in the Flemish region of Belgium to 120 μg Hb/g in England, Wales, and Northern Ireland. The majority of countries (20 out of 30) adopted cut-offs in the 10–30 μg Hb/g range, with the majority of screening programs utilizing the OC-Sensor FIT diagnostic kit.^3^ However, the Sentinel Diagnostics test which was used for evaluating diagnostic performance in the BLITZ study, was also used in several countries, including Belgium, the Czech Republic, the Netherlands and Sweden.

**Table 2:** Sensitivity and specificity of a quantitative FIT (Sentinel Diagnostics) at positivity thresholds used in FIT-based screening programs in various countries/regions.

Threshold (μg/g)	Country, regions	Test manufacturer^a^				Positivity rate (95% CI)	Sensitivity (95% CI)			Specificity (95% CI)
		OC	SD	AP	MM		CRC	APCL	AN	no AN
8.5	Belgium, Flanders		x			17.6 (16.7, 18.5)	98.1 (90.1, 100.0)	45.5 (41.9, 49.0)	48.9 (45.4, 52.4)	86.3 (85.5, 87.2)
10	Austria, Burgenland	x				15.0 (14.2, 15.8)	96.3 (87.3, 99.5)	41.4 (37.9, 45.0)	45.0 (41.6, 48.5)	88.8 (88.0, 89.5)
	Brazil, São Paulo	x								
	Canada, British Columbia	x								
	Russia, multiple regions	*FIT of choice*								
15	Canada, Northwest Territories	x				10.8 (10.1, 11.5)	90.7 (79.7, 96.9)	33.5 (30.2, 37.0)	37.3 (33.9, 40.7)	92.5 (91.8, 93.1)
	Czech Republic	x	x							
	Israel, national	x								
	Latvia, national	x								
	Norway, national	x								
	Switzerland, Vaud	x								
20	Australia	x				8.8 (8.2, 9.5)	88.9 (77.4, 95.8)	29.7 (26.5, 33.1)	33.6 (30.4, 37.0)	94.3 (93.7, 94.8)
	Canada, multiple provinces^c^	x								
	Canada, Newfoundland + Labrador			x						
	Denmark, national	x								
	Italy, national	x								
	Japan^d^	x		x						
	Mexico	x								
	Romania, South Muntenia	x								
	Slovenia, national	x								
	Spain, multiple regions^e^	x								
	Taiwan, national^b^	x								
	United States	x								
25	Finland, national	x				7.6 (7.0, 8.2)	87.0 (75.1, 94.6)	26.9 (23.8, 30.2)	30.8 (27.7, 34.1)	95.3 (94.8, 95.8)
30	France, national	x				6.7 (6.2, 7.3)	85.2 (72.9, 93.4)	24.7 (21.7, 27.9)	28.6 (25.6, 31.9)	96.0 (95.5, 96.5)
	Canada, Ontario	x								
	Taiwan, national^b^				x					
40	New Zealand, national	x				5.7 (5.1, 6.2)	81.5 (68.6, 90.7)	21.6 (18.7, 24.6)	25.5 (22.5, 28.6)	96.8 (96.4, 97.2)
	Sweden, national, women		x							
45	Irish Republic, national	x				5.2 (4.7, 5.8)	79.6 (66.5, 89.4)	20.6 (17.8, 23.7)	24.5 (21.6, 27.6)	97.2 (96.7, 97.6)
47	The Netherlands, national		x			5.1 (4.6, 5.6)	79.6 (66.5, 89.4)	20.0 (17.2, 23.0)	23.9 (21.0, 27.0)	97.2 (96.8, 97.6)
80	United Kingdom, Scotland				x	3.7 (3.3, 4.1)	75.9 (62.4, 86.5)	15.7 (13.2, 18.5)	19.7 (17.0, 22.5)	98.3 (98.0, 98.6)
	Sweden, national, men		x							
120	United Kingdom, England, Wales, Northern Ireland	x				2.6 (2.3, 3.0)	55.6 (41.4, 69.1)	12.2 (10.0, 14.7)	15.0 (12.7, 17.7)	98.9 (98.7, 99.2)

Other abbreviations: AN, advanced neoplasia; APCL, advanced precancerous lesions; CRC, colorectal cancer; CI, confidence interval.

a OC, OC-Sensor; SD, Sentinel Diagnostics; AP, Alfresa Pharma; MM, Minaris Medical Co. Ltd (HM-JACKarc).

b FIT selection varied by municipality, based on local procurement procedures and institutional considerations.^12^

c Regions: Manitoba, New Brunswick, Prince Edward Island, Saskatchewan.

d Test choice varied by prefecture, with OC-SENSOR used in Tokyo and 29 other prefectures, and Alfresa Pharma in Aomori and 9 others; the most common cut-off was 20 μg Hb/g, though this varied by prefecture.

e Regions: Barcelona, Catalonia, Galicia, Basque Country, Valencia.

In the BLITZ study, the FIT positivity strongly declined from 17.6% (95% CI: 16.7–18.5) at the 8.5 μg Hb/g cutoff to 2.6% (95% CI: 2.3–3.0) at the 120 μg Hb/g cutoff. Sensitivities likewise strongly declined, whereas specificity strongly increased with increasing cutoffs.

#### FIT performance at low cut-off levels (<20 μg Hb/g)

**Fig. 2:**
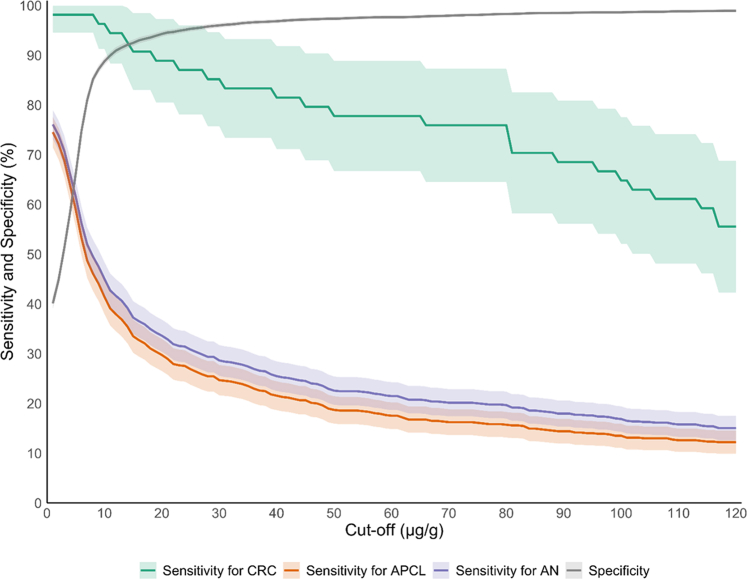
Sensitivity and specificity trends for CRC, APCL, and AN according to FIT cutoff, continuously varied from 2 to 120 μg/g. Abbreviations: AN, advanced neoplasia; APCL, advanced precancerous lesions; CRC, colorectal cancer; FIT fecal immunochemical test.

Cut-off levels <20 μg Hb/g, which were employed in 10 countries, exhibited the highest FIT sensitivity, ranging from 98.1% (95% CI: 90.1–100.0) of all CRC at the lowest cut-off level analyzed, 8.5 μg Hb/g (as used in Belgium's Flemish region) to 90.7% (95% CI: 79.7–96.9) at a threshold of 15 μg Hb/g (used in Canada's Northwest Territories, Israel, Norway, and Switzerland). Corresponding sensitivities for APCL ranged from 45.5% (95% CI 41.9–49.0) to 33.5% (30.2–37.0). However, specificities were lower than 90% for the 8.5 and 10 μg Hb/g cutoff (86.3 and 88.8), and still only 92.5% for the 15 μg Hb/g cutoff (Table 2, Fig. 2).

#### FIT performance at intermediate cut-off levels (20–30 μg Hb/g)

At intermediate cut-offs from 20 to 30 μg Hb/g, which were employed in the majority of countries, sensitivities ranged from 88.9% to 85.2% for CRC and from 29.7 to 24.7% for APCL, while specificity was around 95% (ranging from 94.3% at the 20 μg Hb/g cutoff to 96% at the 20 μg Hb/g cutoff (Table 2, Fig. 2).

#### FIT performance at higher cut-off levels (≥40 μg Hb/g)

At cutoffs above 30, sensitivity further substantially declined, from 81.5% for CRC and 21.6% for APCL at the 40 μg Hb/g cutoff, employed in New Zealand and Sweden for women, to 55.6% and 12.2%, respectively, at the 120 μg Hb/g cutoff, employed in England, Wales, and Northern Ireland. At the same time a further modest increase in specificity from 96.8% to 98.9% was observed (Table 2, Fig. 2).

### Sex- and age-specific FIT performance

Further analyses by sex show analogous variations of positivity rate, sensitivities and specificity with FIT cutoffs even though positivity rates and sensitivities tended to be higher among men (Supplementary Table S1, Supplementary Figure S1). At the cut-off range from 8.5 to 120 μg Hb/g, the positivity rate ranged from 13.6% to 2.1% in women and from 21.0% to 3.1% in men. Similarly, FIT sensitivity for CRC ranged from 95.2% to 52.4 in women and from 100% to 57.6 in men. Like specificities, PPVs also increased with increasing cutoffs among both women and men, but they were consistently higher among men than among women given the higher prevalences of advanced neoplasms among men.

Age-specific analyses revealed similar inverse relationships between FIT thresholds and positivity rate and sensitivity, and similar positive relationships between specificity and PPV in both age groups, 50–59 and 60–79 years. At cut-offs from 8.5 to 120 μg Hb/g, FIT sensitivity for CRC ranged from 100.0% to 40.0% in younger participants (50–59 years) and from 97.4% to 61.5% in the 60–79-year-old group. Similarly, the positivity rate ranged from 14.4% to 1.9% in the younger participants and from 19.9% to 3.3% in the older group, respectively. The PPV of FIT was notably higher across all cutoffs in the older age group (Supplementary Table S2, Supplementary Figure S2).

### Clinical impact analysis

**Fig. 3:**
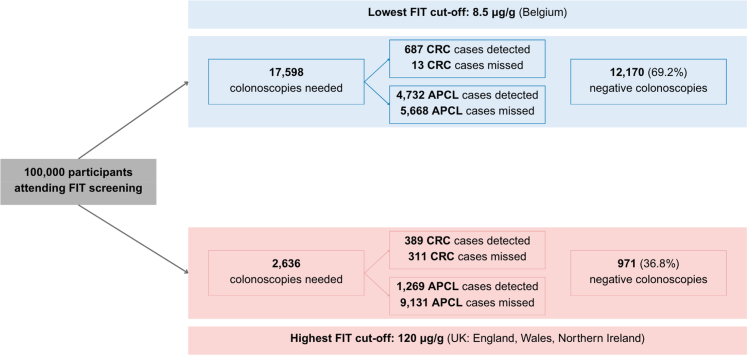
Screening outcomes per 100,000 participants at the lowest and highest FIT threshold. Abbreviations: APCL, advanced precancerous lesions; CRC, colorectal cancer; FIT fecal immunochemical test.

In our clinical impact analysis of a hypothetical population of 100,000 individuals undergoing FIT screening, the lowest threshold of 8.5 μg Hb/g detected nearly all CRC cases, with 687 of 700 (98.1%) identified, alongside 4732 APCL, but required 17,598 colonoscopies, including 12,170 negative procedures with no AN detection. In contrast, at the highest threshold of 120 μg Hb/g, CRC detection declined markedly to 389 cases (55.6%), with 311 missed, and APCL detection reduced to 1269, while colonoscopy demand dropped substantially to 2636 procedures, of which 971 were negative examinations (Fig. 3, Supplementary Table S3).

## Discussion

This study evaluated the diagnostic performance metrics of FIT in the detection of CRC or any advanced neoplasia across varying positivity thresholds currently in use in population-based CRC screening programs in 30 countries, regions, and provinces in five continents. Our findings demonstrate very large variation in sensitivity and specificity of FIT across the applied thresholds, ranging from 98.1% to 55.6% for CRC sensitivity, 48.9%–15.0% for AN sensitivity, and from 86.3% to 98.9% for specificity. Additionally, differences in FIT performance were observed by sex and age, with men and older individuals generally demonstrating higher positivity rates, sensitivity, and positive predictive values than women and younger individuals at equivalent cut-off levels. The results indicate wide variability in diagnostic performance indicators in FIT-based CRC screening programs, offering some explanation for the differences in the real-world implications on CRC incidence and mortality among the included countries.

The need for a balanced compromise between diagnostic accuracy and the operational demands of follow-up colonoscopy often dictate adoption of FIT thresholds in various countries,^3^ with many countries opting for higher thresholds to limit the colonoscopy burden. Nevertheless, an increase in the FIT threshold, while improving specificity and reducing colonoscopy need, results in a decrease in sensitivity, which may compromise the overall effectiveness of CRC screening by missing early cases of cancer or advanced neoplasia.^5^^,^^13^ This trade-off was evident in our analyses, where the highest threshold substantially reduced colonoscopy demand to less than one sixth, but at the expense of missing almost half of the cancers and a substantial proportion of high-risk precancerous lesions.

Conversely, a key limitation of adopting lower FIT thresholds, beyond the strain on colonoscopy capacity, is the increased rate of false positive results.^5^^,^^14^^,^^15^ This results in a higher proportion of unnecessary (negative) colonoscopies, potentially undermining the cost-effectiveness advantages of population-based CRC screening programs. For example, it has been estimated that up to 60–80% of colonoscopies performed for combined indications such as FIT positivity, post-polypectomy surveillance, and clinical symptoms may yield no significant diagnostic findings.^5^ In our study, nearly 70% of follow-up colonoscopies were expected to be negative examinations when applying the lowest FIT cutoff (8.5 μg Hb/g).

Overall, our findings are consistent with prior studies demonstrating strongly varying FIT positivity rates and detection rates of CRC and its precursors across varying hemoglobin thresholds.^6^^,^^9^^,^^16^ For example, a recent analysis from a large-scale screening trial in Norway reported a decrease of the positivity rate from 8.5 to 2.2% if the FIT cutoff of 15 μg Hb/g that was used in the trial had been increased to 120 μg Hb/g,^6^ a decrease in positivity rate similar to the one observed in our study (from 10.8% to 2.6%). However, in this screening trial, like in other screening studies,^7^ results of colonoscopy were available for people with a FIT value above the trial's pre-specified cutoff only. Therefore, only relative sensitivities and specificities for the various cutoffs above the trials' cutoffs could be evaluated. By contrast, availability of colonoscopy results in all study participants enabled derivation of absolute sensitivities and specificities in our study.

Consistent with our findings, previous studies have reported lower sensitivities and higher specificities for FIT in women compared to men, with similar sex differences observed in PPV and positivity rates.^17^, ^18^, ^19^ Some, albeit less consistent variation of sensitivity and specificity has also been observed by age.^18^, ^19^, ^20^ These differences in FIT performance by sex and age have prompted suggestions to further optimize FIT-based screening through the use of age- and sex-specific positivity thresholds.^18^ To our knowledge, only a Swedish program currently implements sex-specific thresholds in a national population screening setting,^21^ while a similar sex-specific protocol has been the focus of an ongoing pilot in Finland since 2019.^22^ However, although the lower threshold for women (40 μg Hb/g) than for men (80 μg Hb/g) in the Swedish program may reduce or overcome sex differences in sensitivity and specificity, it will increase sex differences in PPV (and numbers needed to scope to detect CRC and its precursors), given the higher prevalence of colorectal neoplasms among men than among women.^23^ Further research is needed to evaluate pros and cons of sex-specific positivity thresholds.

Key strengths of our study include that it was conducted in the context of a long-established screening program, and that all participants underwent colonoscopy irrespective of their FIT results. This approach minimizes verification bias and enables a robust and accurate assessment of FIT performance including absolute sensitivities and specificities across varying thresholds. Additionally, our stratified analysis provides valuable insights into variation of FIT performance by sex and age. The inverse relationship between FIT threshold and sensitivity has been well documented, and comparable performance estimates could be derived across the full continuum of possible FIT cut-offs (as shown in our Fig. 2). Our study extends existing evidence and enhances interpretability by translating this principle into a real-world, policy-relevant context. By applying cut-offs currently used in national screening programs across five continents to a single, well-characterized population, we demonstrate the magnitude of variation in diagnostic yield and colonoscopy demand that arises solely from threshold selection.

Several limitations of this study should also be acknowledged. While the Sentinel Diagnostics brand used in this study is employed in some national screening programs, the findings may not be fully generalizable to settings using other FIT brands with different analytical characteristics. Nonetheless, previous direct comparisons of multiple FIT brands in a subsample of the BLITZ study demonstrated that apparent inter-brand variability in diagnostic performance could be minimized by adjusting cut-off thresholds to achieve comparable specificity or positivity rates.^10^ Although the study population was drawn from a screening colonoscopy cohort, which may differ from FIT-screened populations, the standardized colonoscopy reference enables unbiased comparison of diagnostic performance across FIT thresholds, with relative trade-offs remaining informative for screening policy.

We did not perform a formal cost-effectiveness analysis. While such analyses are essential for policy decision-making, they require country-specific cost structures, screening participation patterns, and long-term outcome data that were not available within the scope of this study. However, by systematically quantifying diagnostic performance and colonoscopy demand across a wide range of FIT thresholds, our results provide a robust empirical foundation for future health economic evaluations and optimization of FIT thresholds. Since the estimated PPVs and our clinical impact analysis were based on disease prevalence observed in the BLITZ study, the absolute numbers of detected lesions and required colonoscopies should be interpreted as illustrative, while the relative differences across FIT thresholds are expected to remain informative across populations with differing CRC and APCL prevalence. Similarly, national FIT thresholds are shaped by local epidemiology and health-system constraints. Differences in screening pathways and colonoscopy capacity may further influence the practical implications of adopting specific thresholds. Our analysis does not seek to challenge these context-specific decisions, but rather to provide a standardized benchmarking framework that illustrates how different policy-relevant thresholds translate into diagnostic performance under comparable conditions. Therefore, policymakers may use these estimates to assess how alternative thresholds would affect cancer detection and colonoscopy demand across different capacity scenarios, thereby supporting evidence-informed adjustments as screening programs evolve.

This study was limited to short-term diagnostic outcomes and did not evaluate long-term endpoints such as CRC mortality reduction, which are critical for evaluating the ultimate impact of CRC screening programs. Additionally, although race-specific analyses have been recommended in prior research,^20^ our study population was predominantly white, which precluded stratified analyses by race. This limits the generalizability of our findings to more racially diverse populations.

While we acknowledge that repeated screening rounds may increase the cumulative sensitivity of FIT-based programs, single-round diagnostic performance (used in this study reflecting the immediate diagnostic yield and colonoscopy demand associated with different positivity thresholds) remains a critical determinant of screening effectiveness and provides the necessary foundation for modeling long-term outcomes across multiple rounds.

In conclusion, our study reveals substantial variability in the diagnostic performance of FIT across commonly used thresholds worldwide and reinforces the inherent trade-offs between sensitivity and specificity. These findings emphasize both the potential and the need to optimize FIT-based screening strategies in order to maximize benefits for target populations within diverse healthcare settings. Our findings may provide important information for defining or re-adjusting FIT cutoffs in newly established or existing screening programs. Future modeling studies should evaluate expected long-term effectiveness and cost-effectiveness of FIT-based screening programs using various FIT cutoffs. We hope that our results may inform such studies and thereby contribute to selecting optimal cutoffs for various healthcare settings.

## Contributors

Idris Ola: Methodology, Visualization, Writing—original draft, Writing—review & editing; Teresa Seum: Data curation, Formal Analysis, Methodology, Visualization, Writing—review & editing; Sigrid Carlsson: Writing—review & editing; Michael Hoffmeister: Conceptualization, Writing—review & editing; Hermann Brenner: Conceptualization, Funding acquisition, Methodology, Supervision, Writing—review & editing. All authors have read and approved the submitted version. All the authors had full access and verified the data used in the study.

## Data sharing statement

The data that support the findings of this study are available upon reasonable request.

## Declaration of interests

The authors disclose no conflicts of interest in the conduct of this study.

## References

[1] Ola I., Cardoso R., Hoffmeister M., Brenner H. (2024). Utilization of colorectal cancer screening tests across European countries: a cross-sectional analysis of the European health interview survey 2018-2020. Lancet Reg Health Eur.

[2] Ola I., Cardoso R., Hoffmeister M., Brenner H. (2024). Utilization of colorectal cancer screening tests: a systematic review and time trend analysis of nationally representative data. eClinicalMedicine.

[3] Young G.P., Benton S.C., Bresalier R.S. (2025). Fecal immunochemical test positivity thresholds: an international survey of population-based screening programs. Dig Dis Sci.

[4] Forbes N., Hilsden R.J., Martel M. (2021). Association between time to colonoscopy after positive fecal testing and colorectal cancer outcomes: a systematic review. Clin Gastroenterol Hepatol.

[5] McFerran E., O'Mahony J.F., Naber S. (2022). Colorectal cancer screening within colonoscopy capacity constraints: can FIT-Based programs save more lives by trading off more sensitive test cutoffs against longer screening intervals?. MDM Policy Pract.

[6] Randel K.R., Botteri E., de Lange T. (2025). Performance of faecal immunochemical testing for colorectal cancer screening at varying positivity thresholds. Aliment Pharmacol Ther.

[7] Westerberg M., Eriksson J., Metcalfe C. (2024). Colonoscopy findings after increasing two-stool faecal immunochemical test (FIT) cut-off: Cross-sectional analysis of the SCREESCO randomized trial. J Intern Med.

[8] Brenner H., Tao S. (2013). Superior diagnostic performance of faecal immunochemical tests for haemoglobin in a head-to-head comparison with guaiac based faecal occult blood test among 2235 participants of screening colonoscopy. Eur J Cancer.

[9] Chen H., Werner S., Brenner H. (2017). Fresh vs frozen samples and ambient temperature have little effect on detection of colorectal cancer or adenomas by a fecal immunochemical Test in a colorectal cancer screening cohort in Germany. Clin Gastroenterol Hepatol.

[10] Gies A., Cuk K., Schrotz-King P., Brenner H. (2018). Direct comparison of diagnostic performance of 9 quantitative fecal immunochemical tests for colorectal cancer screening. Gastroenterology.

[11] Niedermaier T., Heisser T., Cardoso R., Hoffmeister M., Brenner H. (2023). Colonoscopy-Ascertained prevalence of advanced Neoplasia according to fecal hemoglobin concentration in a large cohort of fecal immunochemical test-negative screening participants. Ann Intern Med.

[12] Chiu S.Y., Chuang S.L., Chen S.L. (2017). Faecal haemoglobin concentration influences risk prediction of interval cancers resulting from inadequate colonoscopy quality: analysis of the Taiwanese Nationwide Colorectal Cancer Screening Program. Gut.

[13] Allison J.E., Fraser C.G., Halloran S.P., Young G.P. (2014). Population screening for colorectal cancer means getting FIT: the past, present, and future of colorectal cancer screening using the fecal immunochemical test for hemoglobin (FIT). Gut Liver.

[14] Wong M.C., Ching J.Y., Chan V.C. (2015). Factors associated with false-positive and false-negative fecal immunochemical test results for colorectal cancer screening. Gastrointest Endosc.

[15] Amitay E.L., Cuk K., Niedermaier T., Weigl K., Brenner H. (2019). Factors associated with false-positive fecal immunochemical tests in a large German colorectal cancer screening study. Int J Cancer.

[16] Aniwan S., Ratanachu Ek T., Pongprasobchai S. (2017). The optimal cut-off level of the fecal immunochemical test for colorectal cancer screening in a country with limited colonoscopy resources: a multi-center study from Thailand. Asian Pac J Cancer Prev.

[17] Brenner H., Qian J., Werner S. (2018). Variation of diagnostic performance of fecal immunochemical testing for hemoglobin by sex and age: results from a large screening cohort. Clin Epidemiol.

[18] Chen S.L., Hsu C.Y., Yen A.M. (2018). Demand for colonoscopy in colorectal cancer screening using a quantitative fecal immunochemical test and age/sex-Specific thresholds for test positivity. Cancer Epidemiol Biomarkers Prev.

[19] Gies A., Niedermaier T., Alwers E. (2021). Consistent Major differences in Sex- and age-specific diagnostic performance among nine faecal immunochemical tests used for colorectal cancer screening. Cancers (Basel).

[20] Selby K., Levine E.H., Doan C. (2019). Effect of sex, age, and positivity threshold on fecal immunochemical test accuracy: a systematic review and meta-analysis. Gastroenterology.

[21] Ribbing Wilén H., Blom J. (2024). Interval cancer after two rounds of a Swedish population-based screening program using gender-specific cut-off levels in fecal immunochemical test. J Med Screen.

[22] Sarkeala T., Farkkila M., Anttila A. (2021). Piloting gender-oriented colorectal cancer screening with a faecal immunochemical test: population-based registry study from Finland. BMJ Open.

[23] Brenner H., Hoffmeister M. (2024). Making the best use of quantitative fecal immunochemical test results in colorectal cancer screening. J Intern Med.

