# Supplementary material for: Variation in diagnostic performance of fecal immunochemical test (FIT) by cutoffs used in screening programs globally
Source: eClinicalMedicine. 2026 Mar 16;94:103835. doi: 10.1016/j.eclinm.2026.103835 (PMC13011066; doi:10.1016/j.eclinm.2026.103835)
Supplement: Supplementary Appendix [file mmc1.docx]

**Supplementary Materials**

**Supplementary Table 1.** Sex-specific sensitivity and specificity of quantitative FIT at positivity thresholds used in FIT-based screening programs in various countries.

| **Sex** | **Threshold** (µg/g) | **Positivity rate**  (95% CI) | **Sensitivity**  (95% CI) | | | **Specificity** (95% CI) | **PPV**  (95% CI) |
| --- | --- | --- | --- | --- | --- | --- | --- |
|  |  |  | **CRC** | **APCL** | **AN** | **no AN** |  |
| Women  (n=3,815) | 8.5 | 13.6 (12.5, 14.7) | 95.2 (76.2, 99.9) | 38.2 (32.8, 43.8) | 41.8 (36.5, 47.3) | 88.8 (87.7, 89.8) | 3.8 (2.3, 5.8) |
|  | 10 | 11.5 (10.5, 12.5) | 95.2 (76.2, 99.9) | 34.4 (29.2, 39.9) | 38.2 (33.0, 43.7) | 90.9 (89.9, 91.9) | 4.5 (2.8, 6.9) |
|  | 15 | 8.8 (7.9, 9.7) | 90.5 (69.6, 98.8) | 29.3 (24.3, 34.7) | 33.1 (28.1, 38.5) | 93.6 (92.7, 94.4) | 5.7 (3.4, 8.7) |
|  | 20 | 6.9 (6.1, 7.8) | 85.7 (63.7, 97.0) | 26.1 (21.3, 31.3) | 29.9 (25.0, 35.1) | 95.2 (94.4, 95.9) | 6.7 (4.0, 10.4) |
|  | 25 | 5.9 (5.1, 6.6) | 81.0 (58.1, 94.6) | 23.6 (19.0, 28.7) | 27.2 (22.5, 32.3) | 96.1 (95.5, 96.8) | 7.6 (4.5, 11.8) |
|  | 30 | 5.1 (4.4, 5.8) | 81.0 (58.1, 94.6) | 20.4 (16.1, 25.3) | 24.2 (19.7, 29.1) | 96.7 (96.1, 97.3) | 8.7 (5.2, 13.6) |
|  | 40 | 4.2 (3.5, 4.8) | 71.4 (47.8, 88.7) | 17.5 (13.5, 22.2) | 20.9 (16.7, 25.7) | 97.4 (96.9, 97.9) | 9.4 (5.4, 15.1) |
|  | 45 | 3.9 (3.3, 4.5) | 66.7 (43.0, 85.4) | 16.9 (12.9, 21.5) | 20.0 (15.9, 24.7) | 97.6 (97.1, 98.1) | 9.4 (5.2, 15.3) |
|  | 47 | 3.7 (3.1, 4.4) | 66.7 (43.0, 85.4) | 16.2 (12.3, 20.8) | 19.4 (15.3, 24.1) | 97.8 (97.2, 98.2) | 9.8 (5.5, 15.9) |
|  | 80 | 2.9 (2.4, 3.4) | 61.9 (38.4, 81.9) | 12.7 (9.3, 16.9) | 15.8 (12.1, 20.2) | 98.3 (97.9, 98.7) | 11.7 (6.4, 19.2) |
|  | 120 | 2.1 (1.7, 2.6) | 52.4 (29.8, 74.3) | 10.5 (7.4, 14.4) | 13.1 (9.7, 17.2) | 98.9 (98.5, 99.2) | 13.4 (6.9, 22.7) |
|  |  |  |  |  |  |  |  |
| Men  (n=3,583) | 8.5 | 21.0 (19.7, 22.3) | 100.0 (89.4, 100.0) | 50.4 (45.8, 55.1) | 53.8 (49.3, 58.3) | 83.6 (82.3, 84.9) | 4.3 (3.0, 6.0) |
|  | 10 | 18.3 (17.0, 19.5) | 97.0 (84.2, 99.9) | 46.3 (41.6, 51.0) | 49.7 (45.2, 54.2) | 86.4 (85.1, 87.6) | 4.8 (3.3, 6.7) |
|  | 15 | 13.0 (11.9, 14.1) | 90.9 (75.7, 98.1) | 36.4 (32.0, 41.0) | 40.1 (35.7, 44.6) | 91.3 (90.2, 92.2) | 6.4 (4.4, 9.1) |
|  | 20 | 10.7 (9.7, 11.7) | 90.9 (75.7, 98.1) | 32.2 (28.0, 36.7) | 36.2 (31.9, 40.6) | 93.2 (92.3, 94.1) | 7.8 (5.3, 10.9) |
|  | 25 | 9.3 (8.4, 10.3) | 90.9 (75.7, 98.1) | 29.2 (25.0, 33.6) | 33.3 (29.2, 37.7) | 94.4 (93.5, 95.2) | 8.9 (6.1, 12.5) |
|  | 30 | 8.5 (7.6, 9.4) | 87.9 (71.8, 96.6) | 27.6 (23.6, 32.0) | 31.7 (27.6, 36.0) | 95.2 (94.4, 95.9) | 9.5 (6.5, 13.4) |
|  | 40 | 7.2 (6.4, 8.1) | 87.9 (71.8, 96.6) | 24.3 (20.5, 28.6) | 28.6 (24.7, 32.9) | 96.2 (95.4, 96.8) | 11.2 (7.6, 15.7) |
|  | 45 | 6.7 (5.9, 7.5) | 87.9 (71.8, 96.6) | 23.2 (19.4, 27.4) | 27.6 (23.7, 31.8) | 96.6 (95.9, 97.3) | 12.1 (8.3, 17.0) |
|  | 47 | 6.6 (5.7, 7.4) | 87.9 (71.8, 96.6) | 22.6 (18.8, 26.7) | 27.0 (23.1, 31.2) | 96.7 (96.0, 97.3) | 12.3 (8.4, 17.2) |
|  | 80 | 4.5 (3.8, 5.2) | 84.8 (68.1, 94.9) | 17.8 (14.4, 21.6) | 22.3 (18.7, 26.2) | 98.3 (97.7, 98.7) | 17.2 (11.7, 23.9) |
|  | 120 | 3.1 (2.6, 3.7) | 57.6 (39.2, 74.5) | 13.4 (10.4, 16.9) | 16.4 (13.2, 19.9) | 99.0 (98.5, 99.3) | 17.0 (10.5, 25.2) |

Abbreviations: AN, advanced neoplasia; APCL, advanced precancerous lesions; CI, confidence interval; CRC, colorectal cancer; FIT, fecal immunochemical testing; PPV, positive predictive value

**Supplementary Table 2.** Age-specific sensitivity and specificity of quantitative FIT at positivity thresholds used in FIT-based screening programs in various countries.

| **Age group** | **Threshold** (µg/g) | **Positivity rate** (%) | **Sensitivity** (95% CI) | | | **Specificity** (95% CI) | **PPV** (95% CI) |
| --- | --- | --- | --- | --- | --- | --- | --- |
|  |  |  | **CRC** | **APCL** | **AN** | **no AN** |  |
| 50-59 years (n = 3,605) | 8.5 | 14.4 (13.3, 15.5) | 100.0 (78.2, 100.0) | 38.5 (33.2, 44.0) | 41.2 (35.9, 46.6) | 88.0 (86.8, 89.1) | 2.8 (1.6, 4.6) |
|  | 10 | 12.1 (11.0, 13.2) | 100.0 (78.2, 100.0) | 32.7 (27.7, 38.1) | 35.7 (30.6, 41.0) | 90.2 (89.2, 91.2) | 3.4 (1.9, 5.5) |
|  | 15 | 9.0 (8.0, 9.9) | 93.3 (68.1, 99.8) | 27.9 (23.1, 33.1) | 30.7 (25.9, 35.9) | 93.3 (92.4, 94.2) | 4.3 (2.4, 7.2) |
|  | 20 | 7.3 (6.5, 8.2) | 93.3 (68.1, 99.8) | 23.3 (18.9, 28.3) | 26.4 (21.8, 31.4) | 94.7 (93.8, 95.4) | 5.3 (2.9, 8.7) |
|  | 25 | 6.3 (5.5, 7.1) | 86.7 (59.5, 98.3) | 21.2 (16.9, 26.0) | 24.1 (19.6, 28.9) | 95.6 (94.8, 96.2) | 5.7 (3.1, 9.6) |
|  | 30 | 5.5 (4.7, 6.2) | 86.7 (59.5, 98.3) | 20.0 (15.8, 24.7) | 22.9 (18.6, 27.7) | 96.4 (95.7, 97.0) | 6.6 (3.6, 11.0) |
|  | 40 | 4.5 (3.8, 5.2) | 80.0 (51.9, 95.7) | 17.9 (13.9, 22.5) | 20.6 (16.4, 25.2) | 97.2 (96.6, 97.7) | 7.4 (3.9, 12.5) |
|  | 45 | 4.2 (3.6, 4.9) | 80.0 (51.9, 95.7) | 17.3 (13.4, 21.8) | 20.0 (15.9, 24.6) | 97.4 (96.8, 97.9) | 7.8 (4.1, 13.3) |
|  | 47 | 4.1 (3.5, 4.8) | 80.0 (51.9, 95.7) | 17.0 (13.1, 21.5) | 19.7 (15.6, 24.3) | 97.5 (97.0, 98.1) | 8.1 (4.3, 13.7) |
|  | 80 | 3.0 (2.4, 3.5) | 73.3 (44.9, 92.2) | 14.5 (10.9, 18.8) | 17.1 (13.3, 21.5) | 98.5 (98.0, 98.9) | 10.2 (5.2, 17.5) |
|  | 120 | 1.9 (1.5, 2.4) | 40.0 (16.3, 67.7) | 10.3 (7.2, 14.1) | 11.6 (8.4, 15.5) | 99.1 (98.7, 99.4) | 8.7 (3.3, 18.0) |
|  |  |  |  |  |  |  |  |
| 60-79 years (n = 3,793) | 8.5 | 19.9 (18.6, 21.1) | 97.4 (99.9, 86.5) | 50.7 (55.4, 45.9) | 54.5 (59.0, 49.9) | 84.7 (85.9, 83.4) | 4.9 (3.5, 6.7) |
|  | 10 | 17.3 (16.1, 18.5) | 94.9 (99.4, 82.7) | 48.0 (52.7, 43.2) | 51.8 (56.3, 47.2) | 87.3 (88.4, 86.1) | 5.5 (3.9, 7.6) |
|  | 15 | 12.6 (11.5, 13.7) | 89.7 (97.1, 75.8) | 37.7 (42.4, 33.2) | 42.0 (46.5, 37.5) | 91.6 (92.6, 90.6) | 7.3 (5.2, 10.0) |
|  | 20 | 10.2 (9.2, 11.1) | 87.2 (95.7, 72.6) | 34.5 (39.2, 30.1) | 38.8 (43.4, 34.4) | 93.9 (94.7, 93.0) | 8.7 (6.1, 12.0) |
|  | 25 | 8.7 (7.8, 9.6) | 87.2 (95.7, 72.6) | 31.1 (35.7, 26.8) | 35.7 (40.2, 31.4) | 95.1 (95.8, 94.3) | 10.2 (7.2, 13.9) |
|  | 30 | 8.0 (7.1, 8.8) | 84.6 (94.1, 69.5) | 28.2 (32.6, 24.0) | 32.8 (37.2, 28.6) | 95.6 (96.3, 94.9) | 10.9 (7.6, 15.0) |
|  | 40 | 6.7 (5.9, 7.5) | 82.1 (92.5, 66.5) | 24.3 (28.6, 20.4) | 29.0 (33.3, 25.0) | 96.5 (97.1, 95.8) | 12.5 (8.7, 17.3) |
|  | 45 | 6.2 (5.4, 7.0) | 79.5 (90.7, 63.5) | 23.2 (27.4, 19.3) | 27.8 (32.0, 23.8) | 96.9 (97.5, 96.3) | 13.2 (9.1, 18.2) |
|  | 47 | 6.1 (5.3, 6.8) | 79.5 (90.7, 63.5) | 22.3 (26.5, 18.5) | 26.9 (31.1, 23.0) | 97.0 (97.5, 96.3) | 13.5 (9.3, 18.6) |
|  | 80 | 4.4 (3.7, 5.0) | 76.9 (88.9, 60.7) | 16.6 (20.4, 13.2) | 21.5 (25.5, 17.9) | 98.1 (98.5, 97.6) | 18.1 (12.5, 24.8) |
|  | 120 | 3.3 (2.7, 3.9) | 61.5 (76.6, 44.6) | 13.6 (17.2, 10.6) | 17.5 (21.2, 14.2) | 98.8 (99.1, 98.3) | 19.2 (12.7, 27.2) |

Abbreviations: AN, advanced neoplasia; APCL, advanced precancerous lesions; CI, confidence interval; CRC, colorectal cancer; FIT, fecal immunochemical testing; PPV, positive predictive value

**Supplementary Table 3.** Screening outcomes per 100,000 participants based on FIT test performance.

| **Threshold** (µg/g) | **Colonoscopies needed** (N) | **CRC** | | **APCL** | | **AN** | | **Negative colonoscopies** (N) |
| --- | --- | --- | --- | --- | --- | --- | --- | --- |
|  |  | **Detected** (N) | **Missed** (N) | **Detected** (N) | **Missed** (N) | **Detected** (N) | **Missed** (N) |  |
| **Population total** (reference) |  | **700** | **-** | **10,400** | **-** | **11,100** | **-** | **88,900** |
| 8.5 | 17,598 | 687 | 13 | 4,732 | 5,668 | 5,428 | 5,672 | 12,170 |
| 10 | 14,937 | 674 | 26 | 4,306 | 6,094 | 4,995 | 6,105 | 9,942 |
| 15 | 10,786 | 635 | 65 | 3,484 | 6,916 | 4,140 | 6,960 | 6,646 |
| 20 | 8,778 | 622 | 78 | 3,089 | 7,311 | 3,730 | 7,370 | 5,049 |
| 25 | 7,585 | 609 | 91 | 2,798 | 7,602 | 3,419 | 7,681 | 4,166 |
| 30 | 6,721 | 596 | 104 | 2,569 | 7,831 | 3,175 | 7,925 | 3,547 |
| 40 | 5,662 | 571 | 130 | 2,246 | 8,154 | 2,831 | 8,270 | 2,831 |
| 45 | 5,189 | 557 | 143 | 2,142 | 8,258 | 2,720 | 8,381 | 2,469 |
| 47 | 5,126 | 557 | 143 | 2,080 | 8,320 | 2,653 | 8,447 | 2,474 |
| 80 | 3,675 | 531 | 169 | 1,633 | 8,767 | 2,187 | 8,913 | 1,489 |
| 120 | 2,636 | 389 | 311 | 1,269 | 9,131 | 1,665 | 9,435 | 971 |

Abbreviations: AN, advanced neoplasia; APCL, advanced precancerous lesions; CRC, colorectal cancer


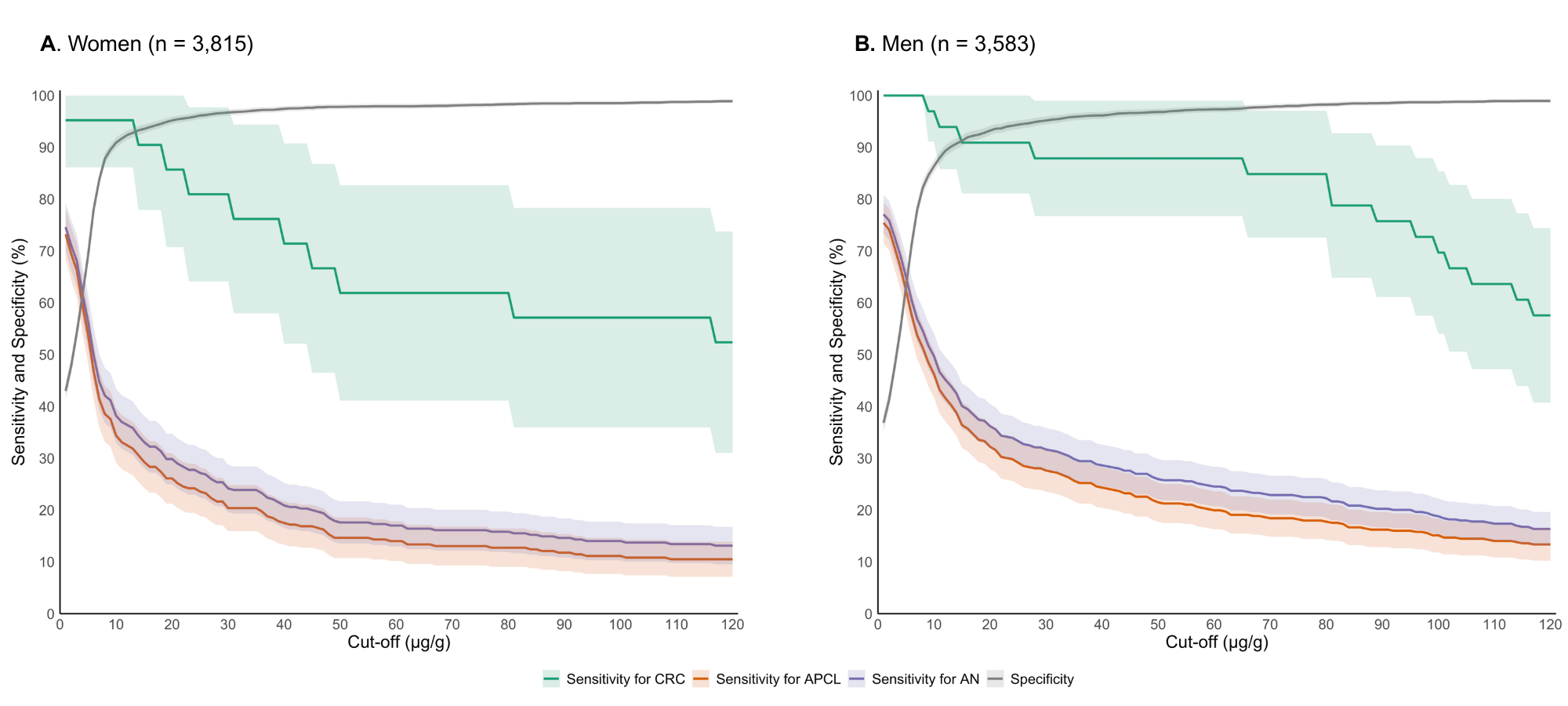


**Supplementary Figure 1.** Sensitivity and specificity trends for CRC, APCL, and AN in female (**A**) and male (**B**) participants according to FIT cutoff, continuously varied from 2 to 120 µg/g.

Abbreviations: APCL, advanced precancerous lesions; AN, advanced neoplasia; CRC, colorectal cancer; FIT, fecal immunochemical testing


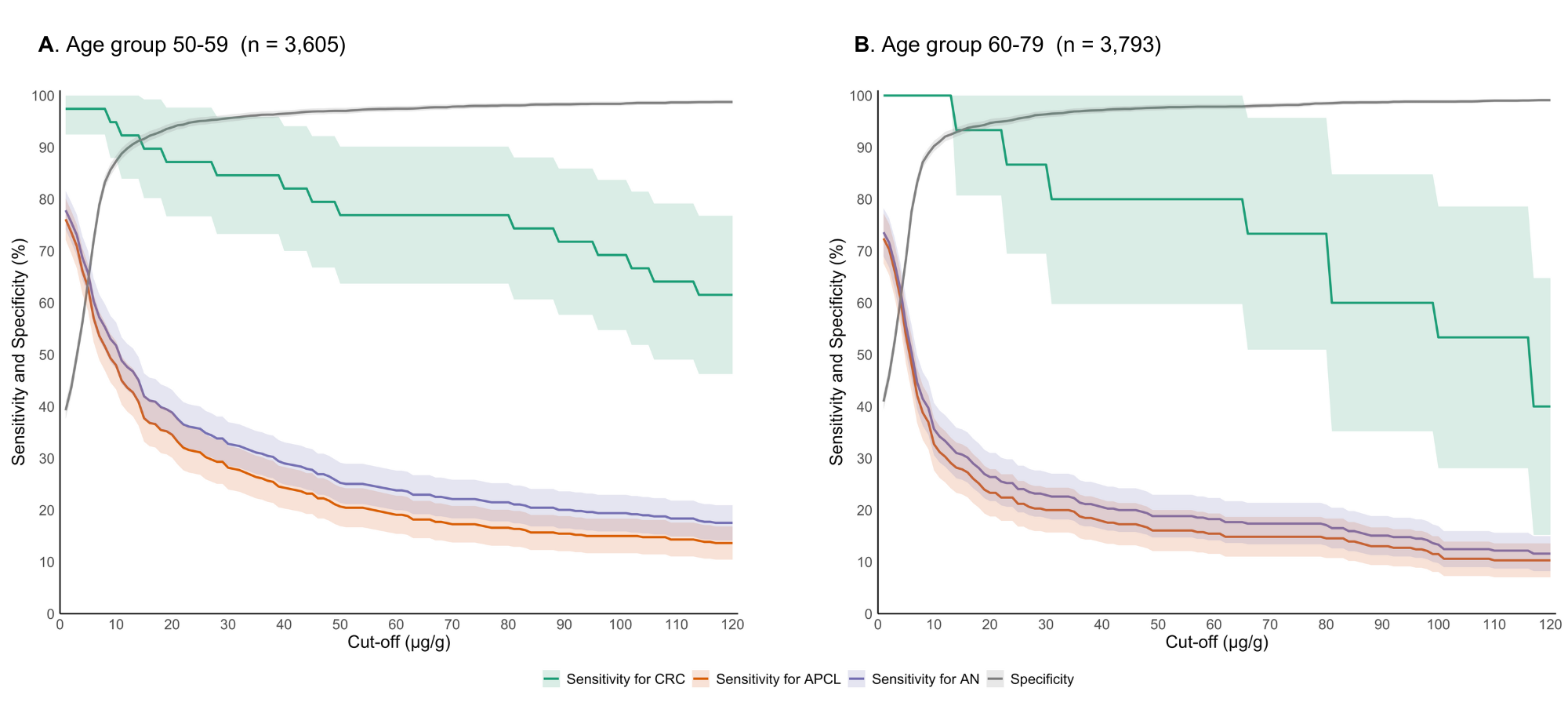
**Supplementary Figure 2.** Sensitivity and specificity trends for CRC, APCL, and AN by age-groups 50-59 years (**A**) and 60-79 years (**B)** according to Sentinel cutoff, continuously varied from 2 to 120 µg/g.

Abbreviations: APCL, advanced precancerous lesions; AN, advanced neoplasia; CRC, colorectal cancer; FIT, fecal immunochemical testing
